# Challenges and opportunities for Iranian global health diplomacy: lessons learned from action for prevention and control of noncommunicable diseases

**DOI:** 10.1186/s12961-021-00800-3

**Published:** 2021-12-24

**Authors:** Mohsen Asadi-Lari, Ahmad Ahmadi Teymourlouy, Mohammadreza Maleki, Leila Eslambolchi, Mahnaz Afshari

**Affiliations:** 1 Department of Epidemiology, School of Public Health, Iran University of Medical Sciences, Tehran, Iran; 2 Department of Health Service Management, School of Health Management and Information Sciences, Iran University of Medical Sciences, Tehran, Iran; 3 Health Management and Economics Department, School of Public Health, Tehran University of Medical Sciences, Tehran, Islamic Republic of Iran; 4 Social Determinants of Health Research Center, Saveh University of Medical Sciences, Saveh, Iran

**Keywords:** Global health diplomacy, International cooperation, Noncommunicable diseases, Policymaking

## Abstract

**Background:**

The steady rise in noncommunicable diseases (NCDs) worldwide has been a key global health challenge. Governments have the primary responsibility for taking action to prevent and control NCDs. Given the growing importance of globalization of healthcare as well as the increasing use of soft power, governments need to identify challenges and opportunities to enhance global health diplomacy (GHD) for NCD prevention and control. The purpose of this qualitative research was to explain the challenges and opportunities of GHD for NCDs in Iran.

**Methods:**

This study was conducted in 2020 using a qualitative approach and through in-depth, semi-structured interviews with 21 experts and specialists in related fields such as health policy, healthcare management, epidemiology and other related specialties. The participants were selected from all levels of diplomacy, including global, regional and national levels, with at least 3 years of experience in managerial, executive and scientific activities. Data analysis was performed by content analysis with an inductive approach. Data were analysed using inductive content analysis.

**Results:**

The identified challenges were categorized into five main themes, including content challenges, structural challenges, process challenges, governance challenges and cultural challenges. Opportunities extracted from the interviews were also categorized into four main themes, including strong political will, utilizing the capacity of nongovernmental organizations (NGOs), multisectoral collaborations and a well-developed health system.

**Conclusions:**

NCD prevention and control requires a multilateral collaboration-based solution. Recognition of the challenges and opportunities in GHD can help draw significant lessons for building the necessary capacities and implementing more effective policies to prevent and control NCDs.

## Background

The steady rise in noncommunicable diseases (NCDs) worldwide has been a key global health challenge [1]. Not only are NCDs a major public health problem, but they also undermine socioeconomic development, especially in low- and middle-income countries [2]. NCDs are the leading causes of death and disability worldwide. In developing countries, the rapidly growing burden of NCDs will have significant social, economic and health consequences [3]. In Iran, NCDs account for more than 82% of the total burden of diseases. According to the Global Burden of Disease Study, mortality from NCDs in Iran has steadily increased from 50% in 1990 to 82% in 2017 [4, 5]. Years of life lost (YLLs) from NCDs increased by 16% from 1990 to 2013 and decreased by 4% from 2013 to 2017 [4].

NCDs are directly related to the incidence of common risk factors such as tobacco use, poor diet and physical inactivity which are preventable. These diseases have become more prevalent as a result of lifestyle changes brought about by industrialization [6]. According to WHO, NCDs kill about 41 million people each year and account for 71% of all deaths globally, more than 15 million of which are premature deaths [7]. Studies have shown that NCDs can be prevented by managing their risk factors. Meanwhile, issues of poverty reduction, access to medicines, and lifestyle changes are also important considerations [8]. A cost-effective and sustainable way to control NCDs is to reduce the risk factors associated with these diseases [9].

Governments have the primary responsibility for taking action to prevent and control NCDs. These measures should create an environment that promotes equity in interventions that aim to reduce unhealthy diets (high in sugar, fat and sodium) and physical inactivity for all age groups. Childhood obesity is a serious and growing problem, which must be tackled along with the social and economic factors that contribute to it. There is also evidence of the role of air pollution as a result of urbanization in the rise of NCDs [10, 11].

The health sector in most countries is primarily focused on healthcare services and rarely on social health factors. As a result, the potential for multisectoral collaboration (government, private sector and civil society) remains untapped, especially in many low- and middle-income countries [12]. In addition, engagement of non-health sectors to reduce NCD risk factors has received little attention. Capacities for initiating and strengthening intersectoral action for NCD prevention are often weak. Most interventions for the entire population should occur outside the healthcare sector, and require support from the public sector, civil society, media and nongovernmental organizations (NGOs) to succeed [13].

Having recognized the importance of NCDs, the international community and governments have taken specific steps to address them through legal frameworks, policies and strategies. The latest example is the 2030 Agenda for Sustainable Development, which was approved by heads of state in September 2015. Goal 3 seeks to “ensure health and well-being for all, at every stage of life” [14]. However, there are challenges along the way. A major challenge is the complexity of these diseases, including the fact that NCDs are caused by many risk factors and not all NCDs are preventable. In light of these complexities, it is difficult to specify targets and fund the necessary actions to meet them. The global response to NCDs should focus on generating multisectoral evidence of transnational factors that contribute to the rise of NCDs and the potential impact of policies proposed to control them [15].

Because of the importance of the NCD issue and the need to respond to general health policies and ensure coherence in decision-making, implementation and monitoring, and evaluation of all activities, in 2014 the Iranian Ministry of Health and Medical Education established a national committee for the prevention and control of NCDs and related risk factors. This committee is responsible for planning, prioritizing, monitoring and evaluating all measures related to the control of NCDs and their risk factors, in the form of a comprehensive and national document and in line with the legal obligations of the Islamic Republic of Iran at the national and international levels. Also, the High Council for Health and Food Security has been tasked with approving multisectoral executive policies to ensure the health of citizens, in line with the objectives of the fourth and fifth development plans. The council worked with the national committee for NCD prevention and control to pursue key goals in multisectoral programmes to combat NCDs. These goals are part of the international obligations of the Islamic Republic of Iran in the form of the “National Action Plan for the Prevention and Control of NCDs and Related Risk Factors” [16].

The purpose of this document is to facilitate and accelerate the achievement of indicators in the field of prevention and control of NCDs through health-oriented measures in the agreed areas. This document addresses issues related to health diplomacy in the field of NCDs, including the prevention of harmful health interventions and the implementation of laws and regulations related to the health appendix, and the establishment of a health secretariat in the office of ministers or heads of organizations to continue health-oriented activities and strengthen multisectoral measures. Iran is one of the countries that has performed best in the prevention and management of NCDs worldwide, and in 2018, WHO selected Iran as one of the first countries to take action against NCDs and introduced it to the world. In fact, Iran’s relations with other countries for scientific exchanges, consensus and medical cooperation have intensified, and Iran is experiencing a period of close and intensive cooperation with WHO [17].

Over the past 15 years, in Iran, due to the multilateral sanctions of Western and American countries, many economic and pharmaceutical challenges have been created, and Iran’s access to markets for goods, services and capital have been limited. Economic sanctions limit a country’s potential growth in both human capital and natural resources. Raw materials for Iran’s industry and consumer goods need to grow; Inputs previously produced through sound trade agreements with the United States and its European allies are no longer available. Pre-sanction imports from the United States to Iran in 1994 were worth US$ 328 million. Current imports barely reached US$ 32 million a year [18]. These sanctions include medicine and equipment. For example, sanctions have reduced Iran's access to cancer medicines. Also, sanctions have disrupted health services through transportation complexity, currency transfers or money shortages and caused a medical crisis due to a shortage of narcotics [19, 20].

In this context, the use of global health diplomacy (GHD) as a corrective action in multilateral and international negotiations is very important and vital, and intervention policies in negotiations should lead to health. GHD is also a means of preserving countries in the global community that is increasingly building reciprocal relationships. In addition, GHD provides a very important opportunity to build bridges and cooperation between governments and nongovernmental organizations (NGOs) to improve public health. GHD is the preferred method of interaction between stakeholders involved in public health and policy to cooperate and resolve disputes, improve health systems and to protect the right to health of vulnerable individuals [21]. Overcoming these challenges posed by sanctions is not possible without resolving international disputes and consulting with other countries through health diplomacy, known as “soft power”. This power can play an important role as one of the main players in the field of removing international health restrictions in Iran [22, 23].

Given the intricate foreign policy context and the complex nature of NCDs, addressing these diseases at the global and national levels requires a great deal of coordination, strategizing and planning. Moreover, governments should mobilize their resources to achieve their goals through cooperation with other countries and international and regional organizations [22, 24, 25]. In this regard, all challenges, factors and strategies influencing GHD for NCD prevention and control should be identified. This qualitative research aims to explain the challenges and opportunities of Iran’s GHD for NCDs.

## Methods

This study was conducted in 2020 using an inductive approach to qualitative data analysis. Data were collected through in-depth, semi-structured interviews. The participants were selected from key informants with extensive knowledge and experience in the phenomenon under study. Accordingly, individuals from WHO (country Office of WHO in the Ministry of Health and Medical Education, WHO Regional Office for the Eastern Mediterranean (EMRO), and WHO headquarters in Geneva), Ministry of Health and Medical Education, International Affairs Department of the Ministry of Health and Medical Education, and selected officials and researchers in the field of NCDs, global health and GHD with at least 3 years of experience in managerial, executive and scientific activities were included. Purposive selection of key informants results in the collection of richer data and a better understanding of the subject matter.

An interview guide was developed based on the objectives of the research and a review of the literature. Three pilot interviews were conducted to optimize the questions, enhance the interviewer’s communication skills and increase the validity of the research. The participants were selected using the snowball sampling technique and continued until data saturation. Interviews were conducted in person at the participants’ workplace or via video calls in Zoom/WhatsApp and took place between October 2019 and July 2020. Each interview lasted between 30 and 90 minutes, with an average duration of 45 minutes. Before the interviews, the participants signed informed consent forms for participating in in-person and online interviews. All the participants agreed to be tape-recorded. Interviewees were identified by the letter “I”. All interviews were transcribed, and the transcripts were submitted to the interviewees to verify and make adjustments if necessary. Transcripts were about 9–18 pages long.

Qualitative content analysis using an inductive approach was employed to analyse the data. This approach avoids predetermined categories and allows insights, categories and themes to emerge from the data. It also reduces the data and provides an accurate description of the topic. Here, the purpose of the inductive analysis was to generate findings from the dominant themes in the data [26, 27]. As part of the process of inductive content analysis, initial coding resulted in 10–15 main categories. Later, these categories were compared, and those that overlapped were linked and grouped under broader categories that represent higher levels of abstraction. These categories can be incorporated into a framework such as an open network, a hierarchy, a temporal order or a causal network [26].

The researchers immersed themselves in the data to obtain new insights. Data analysis began with listening to the interviews and multiple readings of the transcripts to gain a general understanding. Then, the transcripts were reviewed verbatim to extract the codes. This step continued from extraction to the labelling of codes. These codes were then grouped into categories based on their similarities and differences. Finally, evidence from the quoted text was provided for each concept. Data analysis was done in MAXQDA 10.

Measures were taken to increase the validity and reliability of the data, such as having prolonged contact with the research environment, devoting enough time to interviews, having ongoing engagement with the research topic and data, validating the findings with the informants, and exchanging views with peers. Pilot interviews, detailed description of the interview process, constant comparison of the obtained information, and collection of as much information and evidence as possible were some of the strategies employed to ensure the objectivity of the research. An attempt was also made to increase data transferability by enriching the data. Ethical considerations included obtaining approval from the Office of Vice Chancellor for Research at Iran University of Medical Sciences (ethics code: IR.IUMS.REC.1397.438), obtaining informed consent from the interviewees for participation and tape-recording, keeping interviewees’ personal information confidential, and avoiding personal biases in the stages of data collection, analysis and reporting.

## Results

Overall, 21 interviews were conducted with individuals who met the inclusion criteria. The average work experience of the participants was 19 years. The demographic characteristics of the interviewees are provided in Table 1.

**Table 1:** Demographic characteristics of the interviewees

Variable		Frequency	Percentage
Gender	Male	14	66.7
	Female	7	33.3
Education	Masters	3	14.3
	PhD	11	52.4
	MD	4	19.0
	Postdoctoral degree	3	14.3
Work experience	1–10 years	6	28.6
	11–20 years	7	33.3
	20–30 years	7	33.3
	> 30 years	1	4.8
Affiliation	WHO	6	28.6
	NGOs	1	4.8
	Academia	3	14.3
	Ministry of Foreign Affairs	4	19.0
	Ministry of Health and Medical Education	7	33.3

### Challenges

Five main themes were identified as key challenges: content challenges, structural challenges, process challenges, governance challenges and cultural challenges (Table 2).

**Table 2:** Participants’ views on existing challenges

Main themes	Subthemes
Content challenges	Knowledge gap regarding NCDs
	Lack of access to correct information
	The need for stronger declarations of interests
Structural challenges	Organizational conflicts of interest (e.g. conflict of interest of some industries such as tobacco industry with health mission)
	Individual conflicts of interest (profitability of tobacco industry for certain individuals)
	Multisectoral nature of NCDs
	Growing trend of childhood obesity
	Lack of access to essential medicines and services for NCDs
	Financial burden of NCDs, both for individuals/families and for national/health budgets
	Lack of a unified management approach and management language
	Structural weaknesses in developing countries
	Heavy influence of developed nations on the United Nations (UN), which undermines efforts to achieve the ideals set forth by this organization
	Financial aspect of NCDs
	Power and business interests of private companies in the policy environment of NCDs, especially in low- and middle-income countries
	Impact of the private sector on the market and people’s preferences
	Policies made behind closed doors with little transparency
	Low investment by the Ministry of Health and Medical Education as the entity in charge of implementing the Tobacco Control Act compared to the capacity that has been built globally
	Lack of political will to engage all stakeholders in policy development
	Adverse economic conditions
	Food industries have the most complex conflicts of interest due to their special nature
Process challenges	Slow response of the country’s diplomatic apparatus in relation to international organizations
	Appointment of managers based on the wrong principles
	Difficulty in engaging certain agencies
	Failure of health sector outsiders to understand the problem of NCDs
	Different interpretations of the law in sectors other than healthcare
	Plans that are individual-based and are discarded with a change in management
	Weakness of the health sector in health policy-making, health economics and diplomacy
	Lack of transparency in food labelling
	Influence of industries and lobbying by unhealthy commodity industries in the parliament, WHO and other regulatory and legislative bodies
	Unawareness or inability to seize international opportunities
	Poor performance of the country in terms of tobacco control compared to 2030 targets
	Lack of strong NGO presence in actions against NCDs
	Lack of development in the country’s economy, and weak customs and border control
	Passive involvement of the Ministry of Foreign Affairs
	Shortage of financial resources nationwide
	Low priority of NCDs in the national policy agenda
	Lack of coordination between current processes
	Absence of physicians and specialists in the Ministry of Foreign Affairs
	Lack of proper procedure in policy-making nationwide
	Lack of R&D departments in organizations for better identification of best practices
Governance challenges	*Global*
	Soft and superficial approach of the Global Coordination Mechanism
	*National*
	Lack of transparency
	Difficulty deciding on the level of transparency of certain policies such as those related to tobacco use
	Lack of accountability and oversight
	Challenges related to inadequate funds, financing and mobilization of funds to address NCDs
	Disparity between budgets and development plans
	Dependence of the government on the private sector and taxes on unhealthy commodities
	Defects in the law dealing with individuals and organizations that ignore the ban on advertising unhealthy commodities
	Lack of attention to the Development Plan and macroeconomic policies in health policy-making
	Lack of clear mechanisms for identifying and resolving conflicts of interest
	Influence of tobacco companies in health policy
	Resistance of companies, the private sector and executive agencies to the implementation of some documents and laws
	*Health sector*
	Most governments are concerned with primary healthcare or treatment rather than health sector governance
	Lack of strong lobbying or negotiation teams at the level of the Ministry of Health and Medical Education
	Vertical, problem-oriented approach in the health system
	Abolition of the Community Affairs Department, which reduced the follow-up of NCDs and multisectoral action at the local level (provinces, cities and universities)
	Challenges to implementation of the pilot IraPEN [Iran’s Package of Essential NCDs] programme, including the deficiencies of the referral system, the problem of increasing public participation in cities, the high cost of the full package, and the problems caused by sanctions
	Priority of treatment over prevention across the health system
	*Individual*
	Lack of public participation in decision-making and the low public demand for NCD policies
	Democratic governments catering to the wishes of the populace
Cultural challenges	Lack of teamwork
	Low community involvement
	The prevalence of unhealthy diets and sedentary lifestyles
	Resistance to change
	Short-term orientation
	The need for culture-building in line with changes in laws

#### Content challenges

Some of the key challenges facing NCD prevention and control are content-related.

*Gaps in knowledge, especially regarding cardiovascular diseases, is a major challenge given the high burden of these diseases in Iran. Interestingly, this high burden is well-documented, but the knowledge produced is mostly centred around diabetes and cancer. There is a knowledge gap for NCDs that should be addressed*. (I-9)

There is neither enough information nor strong declarations of conflict of interest to prevent powerful companies from influencing policy.

*There’s been a great deal of push to have stronger declarations of conflicts of interest so that these companies don’t sit around the table and write policies for us, or generate the research for us to draw upon. We have to be very transparent about where this evidence comes from, and it’s slowly being exposed. For example, we saw that with Coca-Cola’s attempt to control the narrative. We have to battle these powerful multinational corporations and their influence on policy*. (I-15)

#### Structural challenges

Structural challenges are related to the nature of NCDs, their risk factors and the nature of policy and policy-making. First, the multisectoral nature of NCDs makes them difficult to tackle.

*There are other challenges that are related to the multisectoral nature of NCDs. I mean, we spoke earlier about business factors that indirectly contribute to NCDs, such as exploitation of natural resources, air pollution when it comes to policies that can affect a set of risk factors, how can actors do urban planning for our cities? And how do we make it physically active, friendly? All of this indirectly, of course, affect other sets. So it’s kind of like a water rings, it just evolves and a lot of stakeholders are brought in. And, and that makes it even more I mean, it's not one act or two. But I'm happy to see also that during the last couple of years, there is more attention on NCDs. But I think we’re still struggling to find a way to solve it. To make it that core issue that it needs to be and that it deserves to be about the number of deaths but also the long-term disease and malfunction that brings to society levels*. (I-21)

Organizational and individual conflicts of interest are also among structural challenges.

*And there is also conflict of interest, both individual and organizational. Many industry owners and managers are involved in their industries and wish to influence policies to serve their interests. But some of these conflicts of interest are organizational, including duties and taxes on unhealthy commodities*. (I-3)

Lack of a unified management approach and management language is another structural problem.

*A key intersectoral challenge is the lack of a unified management approach and management language. All policy-makers need to be familiar with public sector management*. (I-7)

The financial aspect of NCDs is also identified as a structural challenge.

*A lot of money is made from NCD risk factors. Billions of dollars annually, as a matter of fact. From food and beverages to tobacco. That is why the private sector doesn’t easily follow scientific evidence from organizations such as WHO or make necessary changes. It’s the duty of governments, especially diplomats, to try to address this issue*. (I-11)

*One of the most important issues, as important as public health, is the financial aspect of NCDs. I mean, the private sector damages public health while paying money for aid. Coca-Cola, for example, contributes to the rise in NCDs, but is also promoting sports activities; it helps organize sports competitions while advertising that, to be healthy, you should play football and drink Coca-Cola!* (I-23)

The financial burden of NCDs is also significant, both for individuals/families and for national/health system budgets. Moreover, poverty is a major contributor.

*Many times, people consume unhealthy food because of their poor financial condition. In some places, fast foods are so commonplace that they’re consumed just because they’re a cheaper alternative to homemade food. You can’t ban fast foods; all you can do is to inform the public*. (I-14)

Policies are mostly made behind closed doors with little transparency.

*More often than not, policies are made behind closed doors where there is little access and transparency*. (I-21)

The influence of private companies and commercial interests in the NCD policy space, especially in low- and middle-income countries, also undermines the progress toward NCD prevention and control.

*There are very powerful commercial interests in the NCD policy space. They push that narrative I just described very hard, but also actively push against any collective multisectoral, multi-stakeholder action*. (I-15)

Moreover, the private sector strongly influences the market and people’s preferences.

*The unfair influence of the private sector on the market and preferences such as tobacco consumption is a major challenge. Smoking has become an economic asset despite being bad for health. When COVID-19 spread, we banned hookah smoking, but many small businesses like cafes and restaurants lost money, which significantly affected the economy. The country cannot and does not have the power to resist all these negative effects. They’re pursuing a policy to ban media campaigns or advertising by the tobacco industry. These are big powerful companies that influence people’s behaviour*. (I-12)

The next important issue is that the UN is heavily influenced by developed countries, which undermines efforts to achieve the ideals set forth by this organization.

*Another point is that, from the very beginning, the UN has been heavily influenced by developed countries. That is, they even put forward an ideal definition of health because these countries are 30 or 40 years ahead of us. You can see the influence of developed countries when you look at UN reports and tools. A simple example is the 1978 conference. These countries came together in Alma-Ata and set targets for health for all that should be achieved by 2000. Many countries did not succeed. They then set eight Millennium Development Goals for 2015. Many countries with widespread poverty, gender inequality and so on didn’t achieve any of these goals by 2015. Many even became worse than before*. (I-7)

In addition, weak structures in developing countries are a challenging issue.

*Developing countries have weak structures and processes. In other words, their competencies are limited. The WHO also highlights the issue of technical assistance, that is, to provide technical assistance instead of financial aid, so that countries develop their own structures and processes*. (I-7)

In terms of tobacco control, not enough infrastructure has been developed as compared to global developments in this field.

*The Ministry of Health is in charge of implementing the Tobacco Control Act, but it hasn’t invested in the necessary infrastructure as much as other countries*. (I-6)

#### Process challenges

There are multiple process challenges to NCD prevention and control. Iran’s diplomatic apparatus has been slow compared to international organizations, and the processes are long and time-consuming.

*Sometimes processes are so drawn out that issues are forgotten before they’re resolved*. (I-17)

Passive involvement of the Ministry of Foreign Affairs is a major process challenge in the action against NCDs.

*Except for the Geneva representative, the Ministry of Foreign Affairs has not been actively involved in the action against NCDs. No one in the Ministry of Foreign Affairs is following up on this issue, and this is a significant weakness*. (I-11)

The absence of a physician or specialist in the Ministry of Foreign Affairs is also challenging.

*We need to have health diplomats, people with a medical degree who can enter the field of diplomacy and serve as liaisons both in Geneva and between Ministry of Foreign Affairs and Ministry of Health*. (I-14)

Sometimes managers are appointed based on the wrong principles. Also, the health sector has been weak in areas of health policy, health economics and diplomacy.

*One problem is our weakness in health policy, health economics and health diplomacy. On the other hand, we’ve closed the doors for multisectoral action by various ministries such as the Ministry of Science and the health sector. And as a result, we’ve made little progress*. (I-7)

Lack of coordination between current processes is one of the challenges to progress on NCDs.

*There are different policies, different measures, different legal instruments, different governance structures, different arrangements, different networks and structures, but they’re not coordinated. That is why we’re now thinking about ways to limit the influence of the private sector and the pharmaceutical industry*. (I-12)

Lack of proper procedure in policy-making across the country is another challenge in this area.

*Policy-making has a proper procedure that, unfortunately, isn’t observed in our country. Sometimes an issue is raised and decided on in a meeting without following the necessary process, which then leads to problems in implementation*. (I-17)

The influence of industries and widespread lobbying by unhealthy commodity industries in the parliament, WHO and other regulatory and legislative bodies are major process challenges.

*Various groups, multinational corporations and the private sector whose interests conflict with public health have massive financial backing and publicity, and virtually try anything to influence policy, from lobbying to spending money on ads and media campaigns. For example, the tobacco and beverage industries practically prevent many laws from passing or from being properly enforced. Therefore, a higher authority beyond mere health institutions such as the WHO and the Ministry of Health should take action*. (I-11)

There are no processes to promote transparent disclosure of conflicts of interest.

*Regarding tobacco use, one of the major concerns that countries have is making sure that manufacturers or countries whose economies depend on tobacco products do not exert influence on policy*. (I-14)

The country’s poor performance in tobacco control is another process challenge.

*We have performed poorly in tobacco control compared to the goals set by the WHO, and that is because sometimes the tobacco industry has more influence than the health sector. We can also see this in several other issues such as tobacco smuggling, employment, and the financial and economic problems we’re currently facing*. (I-6)

There is not enough NGO involvement in NCDs.

*Naturally, NGOs can serve as a liaison for the health sector due to their independence from the government. They can be the voice of the people and the health sector in areas where the Ministry of Health can’t tread. If their numbers get higher, they can be much more effective. They are very few and far between at the moment*. (I-6)

The lack of development of the country’s economy and the weakness in customs control and smuggling indicate wrong processes in this field.

*When the country’s economy is not developed well, customs can’t be controlled and smuggling becomes rampant*. (I-11)

#### Governance challenges

At the level of global governance, the approach of the Global Coordination Mechanism is usually very soft and superficial.

*These global coordination mechanisms are very, very lenient. They’re always focused on voluntary measures and try to avoid mandatory measures. They're very reluctant to require things to happen*. (I-15)

At the national level, there is a lack of transparency regarding interventions.

*We also lack clear, effective interventions; we need to work hard in the public health community to define what works and what doesn’t. And we need to work on that, you know, because even when policy-makers acknowledge the importance of NCDs, they may not know what should be done and how to help communities. Policies won’t be clear, but rather isolated. “Oh, let’s increase taxes on sugary drinks”. But that’s only one small intervention. Our interventions should be more strategic and include several measures that will lead to clear outcomes*. (I-15)

Sometimes it is difficult for policy-makers to decide on the level of transparency of certain policies, including those involving tobacco.

*Transparency is important, and the UN has been trying to webcast and broadcast all its meetings live. But there was concern about the meetings of Parties to the Framework Convention on Tobacco Control that if they are broadcast live, cigarette companies will also have direct access and may try to influence decision-makers. So it was decided that a series of meetings be open but not live, with a half-hour interval in which countries can make a decision and then publish the result*. (I-14)

Lack of accountability and oversight are other governance challenges.

*Nobody asks people why you did this; there is no accountability and no strict monitoring mechanism. The Ministry of Industry, Mine and Trade has an agreement with tobacco suppliers that they should all be licensed, and cigarettes should not be sold to people under the age of 18. However, at least within the jurisdiction of Iran University of Medical Sciences, about 80% of hookah bars do not have a license; no one asks for age verification, and there is not enough oversight and accountability. In some countries, if a retailer sells cigarettes to underage people, it will be fined 500 times the price of the cigarette, which could be up to about a thousand pounds. The question is, does this happen in our country.* (I-17)

Lack of financial resources and financing, instability of financial resources, and lack of resource mobilization in the NCD field is a major challenge, and instability of resources means instability of programmes.

*One of the main problems in achieving NCD prevention and control is the lack of financial resources. In Iran, contrary to what is said, resources are allocated to treatment. To solve this problem, a stable financing system must be created in which each executive identifies the resources it needs to deal with NCDs. The required budget is reviewed annually and allocated by the relevant agencies, and the National Coordination Committee can be asked to monitor how each organization spends its budget on NCDs. Budgets for subsequent years can be allocated according to the report of this committee*. (I-17)

The next important issue in financing is the mobilization of resources.

*Taxes on unhealthy commodities, the most important of which are tobacco and cigarettes, and fines and penalties for traffic and healthcare violations are the main sources of sustainable funding that must be accurately adjusted and properly allocated to the executive bodies involved in NCD prevention and control. Unfortunately, the cigarette tax is very low in Iran, around 4%, which has led to a huge drop in resources. In Turkey, taxes account for 80% of the retail price of a pack of cigarettes*. (I-6)

Another financial challenge is the disparity between budgets and development plans.

*The budget is not based on the development plan. One percent of the country’s GDP is spent on tobacco instead of construction. Each year, about 10 000 billion tomans are spent on smoking, and two to three times that on the treatment of smokers. It must be noted that the money to cover these expenses comes out of people’s pockets*. (I-17)

*We need to make sure that the budget is based on needs and that it protects the people. It must be sustainable and cost-effective. In many countries, the problem is not the amount of money, but how it is spent, which depends on governance arrangements, power relations and bargaining power*. (I-12)

Government dependence on the private sector and taxes on unhealthy commodities are also identified as governance challenges.

*Government revenue may be dependent on taxes generated from cigarette and alcohol sales. So the government faces a dilemma with its competing objectives. On the one hand, it wants to ensure that public health is improved and NCDs are tackled and prevented, while on the other hand, it wants to maintain its revenue streams. Part of these revenues will be dedicated to healthcare or education*. (I-19)

Development plans and macroeconomic policies do not receive enough attention in health policy-making.

*All countries have development plans or macroeconomic policies, but in Iran, these development plans and policies do not have the effect they should have and suffer from weak governance. They aren’t valued enough in the development of other plans. For example, some policies focus on the reduction of national spending from oil revenues and reliance on other revenue sources. In practice, we can increase tobacco tax based on this article, but we’ve seen that it’s been very small*. (I-17)

Health in all policies is among these macroeconomic policies.

*It has been declared by the Supreme Leader that all branches of government should consider health in all policies, especially the Ministry of Health and the executive branch, but this hasn’t been the case in practice*. (I-6)

There are also challenges at the level of health sector governance, including vertical and problem-oriented approaches in the health system, priority of treatment over prevention across the health system and lack of strong lobbying and bargaining teams at the level of the Ministry of Health and Medical Education.

*Most of the actions related to NCDs take place outside the jurisdiction of the Ministry of Health and aren’t regulated by it. In many countries, the Ministry of Health doesn’t even have the power to regulate the private sector. Sometimes, the governing structure of the Ministry of Health itself does not support these policies. There are also no integrated measures within the Ministry of Health*. (I-12)

Governments also face challenges at the level of public participation—that is, low public participation in NCD decision-making and low public demand for NCD policies. Also, in a democratic context, governments cater to the wishes of the populace.

*In a democratic context, governments do anything to get reelected. They don’t want to do things that people don’t want them to do. They don’t want to be unpopular. And at the same time, they have to turn around to businesses that have long-standing connections and play a really important role in the economy and say that we have to regulate you. You have to produce less, sell less, generate less revenue and employ fewer people. So it’s a very, very difficult political situation*. (I-19)

#### Cultural challenges

Cultural changes from eating habits to lifestyles have changed public health.

*Cultural changes, including changes in eating habits, have led to the rise in NCDs around the world. In recent decades, malnutrition, fast food consumption, excessive sugar and salt intake, and tobacco and alcohol consumption have been increasingly threatening public health in different populations worldwide*. (I-13)

Society and its prevailing culture affect people’s health behaviours.

*At the end of the day, we are products of our environment. Sedentary lifestyles are increasing in our region faster than ever before*. (I-21)

There are also other cultural challenges such as lack of teamwork, low community engagement, the popularity of ready meals and the rise in unhealthy diets and sedentary lifestyles that hinder NCD prevention and control.

*Our teamwork is weak. We are used to working in silos. We are still far behind in multisectoral action. Teamwork culture has not been instilled in us. Interaction both between and within sectors is weak. Even in a single office, everybody works for themselves. They don’t come together and use each other’s strengths and abilities. This mentality should be changed early in schools*. (I-16)

*Change itself is challenging. In all areas, NCD control needs change, which isn’t an easy task and can’t be achieved by force*. (I-16)

Passing regulations without people understanding why they should be followed will not be effective, and there is a need to build the necessary culture in line with changing laws.

*I know there are opportunities to exercise in parks, there is consciousness around it, but still, we do not embrace it as the very core of our everyday life. So that’s also a contextual factor in some sense in that we have very clear figures showing that in the eastern Mediterranean region, there is a high level of overweight and obesity, particularly among women, which also indirectly is formed indirectly by some culture or by some tendency to maybe not exercising the same way as males*. (I-21)

To meet the challenges mentioned above, it is important to identify the existing opportunities.

### Opportunities

The opportunities extracted from the interviews are categorized into four main themes, including strong political will, utilizing the capacity of NGOs, multisectoral collaboration and a well-developed health system (Table 3).

**Table 3:** Participants’ views on existing opportunities

Main themes	Subthemes
Strong political will	*International/EMRO region*
	Encouragement of countries by the UN and WHO to commit to reducing NCDs and to help developing countries’ tools and policies
	Creating synergies and aligning the efforts of countries through policies adopted in the international arena and providing advocacy as well as agenda-setting for those policies
	Holding advocacy meetings for WHO representatives, the Speaker of the Parliament and the Chairman of the Parliament’s Medical Commission
	Selection of Iranian EMRO consultants in the area of NCDs
	*National*
	Commitment of the President, and signing of the National Document on Prevention and Control of NCDs
	Existence of an international convention that is sanctioned by the government: the ratification of the WHO Framework Convention on Tobacco Control and related regulations as well as the Protocol to Eliminate Illicit Trade in Tobacco Products
	Creating smoke-free cities, starting with Qom, and using its religious capacity in health diplomacy
	Privatization of the Iranian Tobacco Company
Utilizing the capacity of NGOs	Using popular figures such as actors and artists in NCD-related campaigns
	Allocation of 2% of tobacco taxes to NGOs through legislation
	Taking initiative in creating an NCD network by an NGO
Intersectoral collaboration	Collaboration of the Ministry of Health and Medical Education with the Ministry of Foreign Affairs
	Alignment of goals of the activities of healthcare and sports sectors with NCD prevention and control
	Correspondence with the Ministry of Culture regarding banning of tobacco displays in media
	Compliance with standards by the owners of certain industries to gain a competitive advantage in the global market
A well-developed health system	An advanced health system; a well-developed cascade health system (strong health network)
	The Secretariat for the Health and Food Security Council being located in the Ministry of Health and Medical Education
	The use of a model for intersectoral collaboration and evidence-based public participation in the Health and Food Security Council
	Pursuit of intersectoral collaboration for the implementation of the National Document for Prevention and Control of NCDs by the Health and Food Security Council through agreements with executive bodies and via the health secretariats located in these bodies
	Secretariat of the Provincial and City Health Council
	Annual approval of the list of unhealthy commodities by the Health Council and the prohibition of advertising them

#### Strong political will

At the international and regional levels, there is strong political will to advance Sustainable Development Goals (SDGs), to encourage countries to commit to reducing NCDs and to help develop the tools and policies of countries. Policies adopted in the international arena create synergies and align the efforts of countries’ advocacy and agenda-setting for these policies.

*There has been a great deal of advocacy for the drafting of the document by the Speaker, the President, the Regional Director and the executive branch of Iran as well as the President of WHO. As the issue became more prominent internationally, a movement emerged and spread throughout our country*. (I-6)

*The meeting of WHO officials with our country’s senior officials are much more impactful than when this issue is raised and highlighted at lower levels of management*. (I-4)

The presence of Iranian representatives in the High-Level Meeting of the General Assembly on the Prevention and Control of NCDs is also indicative of strong political will in this area.

*In a commission on NCDs consisting of 20 important figures, Iran had one member as the representative of our region, our former Minister of Health, Dr Hashemi*. (I-13)

At the national level, the president’s commitment increased following the signing of the National Document on Prevention and Control of NCDs. Iran has also become a member of important international conventions such as the WHO Framework Convention on Tobacco Control and the Protocol to Eliminate Illicit Trade in Tobacco Products, which helps build the political will to prevent and control NCDs.

*The Iranian Tobacco Company, which used to be state-owned, is now privatized, and its governance role is diminished*. (I-6)

Another strength nationwide is the creation of smoke-free cities, starting from Qom, and the use of its religious capacity in health diplomacy.

#### Utilizing the capacity of NGOs

There have been significant efforts to use the capacity of NGOs in NCD prevention and control.

*Iran is one of the few countries that discuss NGOs in the Comprehensive Tobacco Control Act. Article 8 of this law allocates 2% of the tobacco tax as an aid to NGOs that are actively working toward tobacco control*. (I-6)

*The initiative of an NGO to create an NCD alliance has been very helpful. NGOs can be the voice of the health sector and the people because due to their independence from the government*. (I-6)

*NGOs are really important because they are somehow—they are the ears on the ground. They are much closer to usually talking about as it is the people living with their cities, they see the needs on the ground, they see the everyday barriers to accessing care to accessing a lifestyle that brings health. And they are also—again, they have those capacities, they are more adaptive because they are so much closer to what we want to affect and who we, at the end of the day, want to help*. (I-21)

#### Intersectoral collaboration

There have been good initiatives to promote intersectoral collaboration for the prevention and control of NCDs.

*The cooperation of the Ministry of Health with the Ministry of Foreign Affairs is a very good and valuable professional relationship with a great deal of mutual trust*. (I-14)

The National Document on Prevention and Control of NCDs facilitates this matter.

*The national NCD document was developed with the participation of all departments and ministries. In fact, it is safe to say that it is the only national document that is signed by all members of the High Council for Health and Food Safety and all ministers, and the memorandum at the beginning of this document commits all ministries to act in accordance with its provisions*. (I-1)

*There is the House of Participation, where the representatives from different unions such as teachers, workers, artists, clerics and others are present. It allows them to have their say about health challenges to the working group that is in the governorate and also follows up on the actions that are taken to address them*. (I-5)

#### A well-developed health system

Iran's Ministry of Health and Medical Education has a strong structure.

*Iran is a great example because a lot of work has been done on NCDs. It is also a unique example because we have the Ministry of Health and Medical Education*. (I-21)

*There’s something special about Iran, which is that it has the Ministry of Health and Education and a very strong and prolific scientific community*. (I-20)

*The Health Transformation Plan is a very good example of that. I mean, it’s led to a decade of reform to try to adapt to a different model of care and to expand coverage to vulnerable communities*. (I-20)

*We have 100 000 healthcare liaisons in the country. These people are the link between families and healthcare units. One in every 50 families is selected as a liaison by the households themselves to provide information to the healthcare unit once a week. This is especially the case in cities, because in villages it’s the health workers that are actively involved. A training programme was created for these liaisons (with the help of UNICEF), and they were trained once a week based on the epidemiological needs of the region; for example, to follow up on people who have been diagnosed with diabetes. These people are trained not only in prevention but also in providing medical assistance to people. The community is also involved. Such community engagement is another example of health diplomacy*. (I-8)

## Discussion and conclusions

The UN Political Declaration was an important step in addressing NCDs as a global health challenge in the twenty-first century, which has undermined socioeconomic development and threatened the achievement of international development goals. NCDs are strongly related to the issue of health security, poverty and lack of natural resources. On the other hand, the relationship between NCDs and SDGs and the unfinished Millennium Development Goals is another factor influencing the NCDs on the global agenda, which should be controlled by cooperation and the adoption of appropriate policies and laws at the national and international levels. In this Declaration, it was reported that one of the main challenges of sustainable development in the twenty-first century is strengthening the health system by providing fair health coverage globally and promoting cost-effective access to the prevention, treatment, care and support of NCDs, especially cancer, cardiovascular disease, chronic respiratory disease and diabetes. Also, another significant SDG is creating or strengthening multisectoral national and international policies for the prevention and control of NCDs [28].

Designing and adopting a global response to the rise of chronic diseases in industrialized and developing countries requires policy-makers to engage in GHD. Global health diplomats need to have a deep understanding of institutional structures and how these relationships work [29]. This qualitative study examined the challenges and opportunities of GHD in policy-making for the prevention and control of NCDs. The identified challenges were divided into five categories, including content challenges, structural challenges, process challenges, governance challenges and cultural challenges. The identified opportunities were also categorized into four main themes, including strong political will, utilizing the capacity of NGOs, intersectoral collaboration and a well-developed health system.

The results of this study showed that the need for stronger declarations of conflicts of interest is one of the most important content challenges. In a study by Westrum, competing stakeholder interests, inappropriate assistance and lack of accountability were identified as the most important barriers to the success of GHD. The rising number of NGOs has increased these conflicts of interest [30]. Countries should raise awareness about NCDs across sectors and address conflicts of interest using a strong advocacy and communication strategy surrounding multisectoral action to prevent these diseases. There should be strategies in place to combat policy interference by various industries that hinders the implementation of NCD prevention measures [31]. For example, some players in the private sector who have no conflict of interest can help build a healthy diet and increase physical activity; limit saturated fats, trans fats, sugars and high salts; increase the availability of healthy and nutritious food choices; and review current market practices. Examples of clear cases for explicit exclusion from such partnerships are alcohol and the tobacco industry [32].

One of the main structural challenges identified in this study is the very complex nature of NCDs, which necessitates the implementation of complex strategies. The most effective public health interventions consist of an evidence-based technical package that combines different actions at different levels that improve together and lead to progress in NCD prevention and control [33]. Therefore, one of the challenges policy-makers face in tackling NCDs is the complexity of these diseases, including the fact that these diseases are caused by many risk factors, are associated with a variety of other factors from global to local, and are not always preventable. Due to these complexities, it is difficult to specify targets and fund the necessary actions to meet them. Therefore, the global response to NCDs should focus on producing multisectoral evidence on transnational factors contributing to the rise of NCDs and the potential impact of policies proposed to control them [15].

In addition, sanctions such as financial, travel and trade restrictions are one of the most fundamental governance challenges [31, 34]. National investments remain low, and not enough funds are mobilized internationally. Many policies have been developed, but structure and resources to implement them are scarce [35, 36]. Resource gaps in many countries can be resolved through new financial mechanisms and more efficient use of existing resources. Many NCD interventions are profitable, including raising taxes on tobacco and alcohol, which simultaneously reduce the risks of NCDs and help minimize inequalities [37, 38].

This study identified several cultural challenges to NCD prevention and control, including lack of teamwork, low community involvement and the prevalence of unhealthy diets and sedentary lifestyles. According to Westrum, cultural awareness is one of the key success factors in GHD, which is defined as awareness and sensitivity to the cultural norms and beliefs of the target populations. Historically, there have been numerous cases in which global health efforts failed due to a lack of sensitivity to the opinions of the target populations. Global health stakeholders must be aware of the cultural norms, beliefs and problems of the target population to ensure their cooperation [30].

The UN developed the Global NCD Action Plan 2013–2020 based on the political commitment of heads of state to prevent and control NCDs. This programme includes nine global NCD targets, for which 19 indicators have been defined, and in 2015, 2017 and 2020, reports were published on monitoring the performance of countries in meeting these targets. According to the 2017 report, Iran succeeded in meeting 15 out of 19 indicators, and WHO identified Iran as one of the leading countries in taking action against NCDs, mainly owing to the measures taken in recent years that were spearheaded by Iran’s Minister of Health. These measures were implemented in accordance with the objectives of the Global NCD Action Plan and within four frameworks for governance, care, prevention and treatment of NCDs: establishment of the National Committee for NCD Prevention and Control at the level of senior officials of the Ministry of Health and Medical Education; membership of a significant number of ministers and other senior officials in this committee; strong political commitment of Iran and significant allocation of funds to control NCDs with the help of the Iranian Parliament; development and ratification of the National NCD Prevention and Control Plan and related (local) action plans and their implementation by all urban and rural healthcare centres across the country; unwavering support for various food-related policies, especially food labels, elimination of industrially produced trans-fatty acids, and reduction of sugar and salt levels in industrial food products as well as salt in bread; other initiatives such as inclusion of mental health interventions and reduction of road accidents and addiction; and promotion of intersectoral collaboration through signing cooperation agreements with other relevant sectors through the Health and Food Security Council, subcommittees and provincial councils.

Intersectoral action refers to “actions undertaken by sectors outside the health sector, possibly, but not necessarily, in collaboration with the health sector, on health or health equity outcomes or the determinants of health or health equity” [39]. These actions include actions within and between sectors, at the local, regional, provincial, national and global levels, which are needed to influence the socioeconomic landscape that enables the health and well-being of the people [40].

A healthy global population is essential to supporting progress in the three pillars of sustainable development: economic growth, social justice and environmental protection. The links highlighted above show that ensuring and promoting health for all depends on integrating NCD prevention and control into sustainable development policies and programmes. By doing so, governments can reduce the negative impacts of unsustainable development measures and reverse the prevalence of NCDs.

## Data Availability

Not applicable.

## Abbreviations

NCDs: Noncommunicable diseases
GHD: Global health diplomacy
UN: United Nations
NGOs: Nongovernmental organizations
NCDA: NCD Alliance
SDGs: Sustainable Development Goals
